# Power analysis to detect treatment effects in longitudinal clinical trials for Alzheimer's disease

**DOI:** 10.1016/j.trci.2017.04.007

**Published:** 2017-05-24

**Authors:** Zhiyue Huang, Graciela Muniz-Terrera, Brian D.M. Tom

**Affiliations:** a MRC Biostatistics Unit, University of Cambridge, UK; b Centre for Dementia Prevention, University of Edinburgh, UK

**Keywords:** Power analysis, Clinical trial, Sample size, Multivariate linear mixed-effects model, Composite score, Alzheimer's disease

## Abstract

**Introduction:**

Assessing cognitive and functional changes at the early stage of Alzheimer's disease (AD) and detecting treatment effects in clinical trials for early AD are challenging.

**Methods:**

Under the assumption that transformed versions of the Mini–Mental State Examination, the Clinical Dementia Rating Scale–Sum of Boxes, and the Alzheimer's Disease Assessment Scale–Cognitive Subscale tests'/components' scores are from a multivariate linear mixed-effects model, we calculated the sample sizes required to detect treatment effects on the annual rates of change in these three components in clinical trials for participants with mild cognitive impairment.

**Results:**

Our results suggest that a large number of participants would be required to detect a clinically meaningful treatment effect in a population with preclinical or prodromal Alzheimer's disease. We found that the transformed Mini–Mental State Examination is more sensitive for detecting treatment effects in early AD than the transformed Clinical Dementia Rating Scale–Sum of Boxes and Alzheimer's Disease Assessment Scale–Cognitive Subscale. The use of optimal weights to construct powerful test statistics or sensitive composite scores/endpoints can reduce the required sample sizes needed for clinical trials.

**Conclusion:**

Consideration of the multivariate/joint distribution of components' scores rather than the distribution of a single composite score when designing clinical trials can lead to an increase in power and reduced sample sizes for detecting treatment effects in clinical trials for early AD.

## 1 Introduction

Much effort has been devoted to developing disease-modifying treatments that intervene in the pathobiologic processes involved in the early stage of Alzheimer's disease (AD). Any therapy that is effective at treating this early manifestation of the dementia process may provide an opportunity for managing the disease while patient function is relatively preserved [1]. Standard instruments used to quantify cognitive and functional decline in AD are relatively insensitive to the changes at early AD [2]. This raises challenges for assessing the early changes in cognition and function across the spectrum of AD [3] and makes detecting treatment effects in clinical trials for early AD even harder [2].

Power analysis is standard when designing clinical trials for detecting treatment effects. Ard *et al.* [4] provide a comprehensive review for clinical trials in AD. Misalignment of the power analysis can lead to possible errors in decisions regarding sample size. Too large samples may waste time, resources, and money and may unnecessarily expose some participants to inferior treatment if a treatment could have been shown to be more effective with fewer participants. Significant underestimation of the sample size may be a waste of time as it would unlikely lead to conclusive findings and therefore be unfair to all participants taking part in the trial. In this article, we are interested in the power/sample size to detect the treatment effects on the component scores in clinical trials for early AD.

In the literature of early AD, many researchers have used composite scores as single endpoints for performing power analysis [4]. A composite score is typically a linear combination of the scores of sensitive instruments. It provides a univariate summary of the component scores, avoids the multiple-hypothesis testing problem when each component score is considered separately, and reduces the impact of measurement error [5]. Furthermore, it may be more sensitive to the cognitive and functional decline than its separate components [6].

The construction of a composite score involves the selection and weighting of the component scores. Typically, the selection of the component scores may be based on a broad literature review regarding sensitivity to decline of candidate components [7], with equal weighting tending to be applied, possibly naively, to the chosen components. However, more statistically driven approaches can be used to derive the weights to construct more sensitive composite scores [2], [6], [8], [9], [10], [11], [12].

We therefore classify the statistical strategies used for the construction of a composite score into two major classes. The first is focused principally on selecting the most informative composite components and using prespecified weights not derived from statistical considerations; for example, Raghavan *et al.* [8] identify the informative component instruments based on standardized mean of 2-year change from baseline for a mild cognitive impairment (MCI) cohort and summed them to create a new composite score. The other is focused on “optimizing” the weights assigned to component scores based on an appropriate optimality criterion and is therefore more data driven; for example, some previous proposals find composite weights, which are sensitive to the clinical decline, by fitting linear mixed-effect models (LMMs) to the longitudinal composite scores [2], [6], [9]. Xiong *et al.* [6] propose composite weights that maximize the probability of observing a decline in one participant over a unit interval of time. Their weights can be considered as a special case of the composite weights proposed by Ard *et al.*, who use the power to detect the time effect in a clinical trial as their criterion and obtain the component weights by maximizing this criterion [2]. Ard *et al.*'s approach is applied to construct a composite atrophy index [9]. Another approach within this class is to base the estimation of the composite weights on a criterion that looks at the mean to standard deviation ratio of change over time [10], [11]. Wang *et al.* [12] propose another composite score construct by using a linear clinical decline equation to select and reweight the component scores simultaneously.

In general, using composite scores as single endpoints may lose information to detect the changes in components [3]; for example, a large change in one component can be masked by small changes on other component scores. Data-driven composite scores have been further criticized [7]. Firstly, they may lose clinical interpretation. It is possible that a clinically meaningful component score has small weights in a data-driven composite score [7]. In addition, they may not be consistent across different data sets. Donohue *et al.* [7] apply cross-validation to quantify the out-of-sample performance of optimal composite scores and conclude that the overall performance of the optimal composite scores is worse than those composite scores derived without optimization.

A limited amount of the literature in AD has considered power analysis with multiple endpoints, although multiple endpoints are commonplace in AD. Under the assumption that the component scores are jointly from a multivariate linear mixed-effects model (MLMM), we compare three approaches with regard to their power to detect the treatment effects on component scores. Two of them are with multiple endpoints, whereas the other is with a single-composite endpoint.

## 2 Methods

### 2.1 MLMM for component scores

Mixed-effect models are from a class of useful statistical models for analyzing longitudinal data [13]. They allow a subset of the regression parameters (random effects) to vary randomly between participants and thereby characterize the natural heterogeneity in the target population in these parameters. Fixed effects are used to refer to the regression parameters, which are fixed but unknown and need to be estimated.

Assuming that all possible covariates are balanced (as would be assumed in a clinical trial through randomization), we model the component scores using an MLMM with a random intercept, fixed time, and time by treatment interaction effects. (The addition of further covariates can be easily incorporated if deemed necessary.) Such a model is able to simultaneously characterize the correlations between the component scores at each time *t* and the correlations across time for each component score.

Let *Y*_*ntj*_ be the *j*-th component score of the *n*-th participant at visit time *t*, where *n* = 1,…,*N*, *t* = 1,…,*T*_*n*_, and *j* = 1,…,*J*. Here, the number of visits *T*_*n*_ is a positive integer depending on the *n*-th participant, and the number of component scores *J* is prespecified. We use a linear function to link the component scores with the mixed effects

$$Y_{ntj}=\beta_{j0}+\gamma_{j}\times \left(\text{Treatment}\times \text{Time}\right)+\beta_{j2}\times \text{Time}+b_{nj}+\varepsilon_{ntj}\text{,}$$

where $\gamma_{j}$ is the *j*-th component treatment effect, *b*_*nj*_ is the random intercept that is unique to the *j*-th component score of the *n*-th participant, and $\varepsilon_{ntj}$ is the random error of the *n*-th participant on the *j*-th component score at time *t*. For each *n*, let *b*_*n*_ = (*b*_*n*1_,…,*b*_*nJ*_)^*T*^ independently follow a multivariate normal distribution with a mean vector 0 and a covariance matrix ∑_*b*_. Here, for any matrix or vector *A*, the matrix *A*^T^ is the transpose of *A*. For each *n* and *t*, further let $\varepsilon_{nt}={\left(\varepsilon_{nt1},\ldots ,\varepsilon_{ntJ}\right)}^{T}$ independently follow a multivariate normal distribution with the mean vector 0 and the covariance matrix $\sum_{\varepsilon }$. For each *n* and *t*, the error $\varepsilon_{nt}$ and the random effects *b*_*n*_ are independent.

For each participant *n* and time *t*, the covariance matrix $\sum_{\varepsilon }$ characterizes the correlation structure between the component scores *Y*_*nt*1_,…,*Y*_*ntJ*_. For each participant *n*, the component scores *Y*_*nt*_ = (*Y*_*nt*1_,…,*Y*_*ntJ*_)^*T*^, *t* = 1,…,*T*_*n*_, are independent of each other through time conditional on the random effect *b*_*n*_, but would be correlated marginally.

We can link the LMM for the composite scores to the MLMM for the components by letting $C_{nt}=\sum_{j=1}^{J}w_{j}Y_{ntj}$, $\alpha_{0}=\sum_{j=1}^{J}w_{j}\beta_{j0}$, $\gamma_{w}=\sum_{j=1}^{J}w_{j}\gamma_{j}$, $\alpha_{2}=\sum_{j=1}^{J}w_{j}\beta_{j2}$, $a_{n}=\sum_{j=1}^{J}w_{j}b_{nj}$, and $\delta_{nt}=\sum_{j=1}^{J}w_{j}\varepsilon_{ntj}$, where *w* = (*w*_1_,…,*w*_*J*_)^*T*^ is the vector of weights for the composite score [2]. The LMM for the composite score of the *n*-th participant at time *t* is therefore

$$C_{nt}=\alpha_{0}+\gamma_{w}\times \left(\text{Treatment}\times \text{Time}\right)+\alpha_{2}\times \text{Time}+a_{n}+\delta_{nt}\text{,}$$

where $\gamma_{w}$ is the treatment effect on composite scores, and for each *n*, the random intercept, *a*_*n*_, follows a normal distribution with mean 0 and variance $\sigma_{a}^{2}=w^{T}\sum_{b}w$, and for each *n* and *t*, the random error, *δ*_*nt*_, follows a normal distribution with mean 0 and variance $\sigma_{\delta }^{2}=w^{T}\sum_{\varepsilon }w$.

### 2.2 Power analysis–hypothesis testing formulations

To detect the treatment effects on component scores, we consider three-hypothesis testing problems and their associated test statistics. Rejecting any of the null hypotheses suggests statistically significant component treatment effects.

The first hypothesis testing problem is to test the null hypothesis of no treatment effect in any of the components against the alternative that there is at least one non-zero treatment effect:

$$H_{0}:\gamma =0\;\text{vs}\;H_{A}:\gamma \neq 0\text{,}$$

where $\gamma ={\left(\gamma_{1},\ldots ,\gamma_{J}\right)}^{\text{T}}$ is the *J*-dimensional vector of treatment effects. The Wald statistic ${\text{Ξ}}_{J}={\hat{\gamma }}^{\text{T}}\sum_{\gamma }^{-1}\hat{\gamma }$ can be used, where $\hat{\gamma }$ is the maximum likelihood estimator (MLE) of γ under the assumption of known covariance matrices for *b*_*n*_ and $\varepsilon_{nt}$, and $\sum_{\gamma }$ is the covariance matrix of $\hat{\gamma }$. It follows that under the null hypothesis of no treatment effect for any of the components that the Wald test statistic will be distributed as a χ^2^ distribution with *J* degrees of freedom, $\chi_{J}^{2}$.

The second hypothesis testing problem considered is for the composite treatment effect, defined as a linear combination of the component treatment effects induced by the weights *w* = (*w*_1_,…,*w*_*J*_)^*T*^. Here, we test the null hypothesis of no composite treatment effect versus the alternative of a composite treatment effect. That is,

$${H^{\prime }}_{0}:\sum_{j=1}^{J}w_{j}\gamma_{j}=0\;\text{vs}\;{H^{\prime }}_{A}:\sum_{j=1}^{J}w_{j}\gamma_{j}\neq 0\text{.}$$

The Wald statistic, here, is ${\text{Ξ}}_{JC}\left(w\right)={\left(w^{\text{T}}\sum_{\gamma }w\right)}^{-1}{\left(w^{\text{T}}\hat{\gamma }\right)}^{2}$, which is distributed as $\chi_{1}^{2}$ under the null, ${H^{\prime }}_{0}$.

The last hypothesis testing problem considers the case in which composite scores are used as single endpoints. It aims to test a single treatment effect on the composite scores

$${H^{\prime \prime }}_{0}:\gamma_{w}=0\;\text{vs}\;{H^{\prime \prime }}_{A}:\gamma_{w}\neq 0.$$

Given the variances $\sigma_{a}^{2}$ and $\sigma_{\delta }^{2}$, let ${\hat{\gamma }}_{w}$ be the MLE of $\gamma_{w}$ and $\sigma_{\gamma }^{2}$ be its variance. We can use the Wald statistic ${\text{Ξ}}_{C}\left(w\right)=\sigma_{\gamma }^{-2}{\hat{\gamma }}_{w}^{2}$, which follows the $\chi_{1}^{2}$ distribution under ${H^{\prime \prime }}_{0}$, to test for this type of treatment effect.

The vector of weights *w* has different meanings under the last two hypotheses testing situations. The weights *w* are on the component treatment effects in the second, whereas the weights *w* reweight the component scores in the third. These testing approaches are equivalent only in the very special case of a linear link function, as is assumed in our setting.

Table 1 summarizes these three-hypothesis testing problem formulations. Under an alternative model, all the test statistics follow a noncentral *χ*^2^ distribution and thereby determine the power to reject the associated null hypothesis. However, using less powerful test statistics will lead to larger sample sizes, which may be judged unethical. In the Supplementary document, we prove that for any given weights *w*, the test statistic Ξ_*JC*_(*w*) is no worse with regards to power than Ξ_*C*_(*w*). The test statistic Ξ_*J*_ does not uniformly outperform either Ξ_*JC*_(*w*) or Ξ_*C*_(*w*) over the range of *w*.

**Table 1:** Summary of the three hypothesis testing formulations to detect treatment effects

	Endpoints		
	Multivariate	Multivariate	Single composite
Statistical model	MLMM	MLMM	LMM
Null hypothesis	γ=0	$\sum_{j=1}^{J}w_{j}\gamma_{j}=0$	$\gamma_{w}=0$
Clinical interpretation	Component treatment effects	Composite treatment effect	Treatment effect on composite scores
Test statistic	Ξ_*J*_	Ξ_*JC*_(*w*)	Ξ_*C*_(*w*)
Null distribution	$\chi_{J}^{2}$	$\chi_{1}^{2}$	$\chi_{1}^{2}$

### 2.3 Power analysis–deriving the parameters required from analysis of MCI participants in Alzheimer's Disease Neuroimaging Initiative

For illustration, we conduct a power analysis for a two-arm randomized AD clinical trial with equal allocation probabilities. The component scores consist of the Mini–Mental State Examination (MMSE), the Clinical Dementia Rating Scale-Sum of Boxes (CDR-SB), and the Alzheimer's Disease Assessment Scale–Cognition Subscale (ADAS-11) scores. We use data extracted from the Alzheimer's Disease Neuroimaging Initiative (ADNI) database (http://adni.loni.ucla.ca) to inform the specification of the various parameters required to perform the power analysis. This data set comprises 927 participants who are at MCI at baseline. The MMSE, the CDR-SB, and the ADAS-11 are recorded biannually for each participant over a total follow-up period of 10 years. To more closely satisfy the normality assumptions for the components in light of potential ceiling effects, we apply the Box-Cox transformation to the data and then rescaled them by their baseline standard deviation; see Supplementary Materials for details. The transformations applied are such that higher values of the transformed components indicate worse cognitive functioning.

We fit the MLMM to the three component scores; see the Supplementary Materials for details on how estimates of the rate of change parameters and the appropriate covariance structures necessary for us to perform the power analysis were obtained. The R function *mlmmm.em()* from the *mlmmm* package [14] was used to compute these estimates. The estimated annual rates of change on the transformed MMSE, the transformed CDR-SB, and the transformed ADAS-11 are 0.079 (95% confidence interval [CI]: 0.064, 0.095), 0.061 (95% CI: 0.045, 0.077), and 0.055 (95% CI: 0.040, 0.069), respectively. These annual rates of change correspond to small rates of change on the original untransformed scale and suggest that there is limited cognitive decline in those with MCI over the follow-up period. The estimated covariance matrices are

$${\hat{\Sigma }}_{\varepsilon }=\left[\begin{array}{ccc} 0.56 & 0.07 & 0.09\\ 0.07 & 0.57 & 0.06\\ 0.09 & 0.06 & 0.44 \end{array}\right]\text{and}{\hat{\Sigma }}_{b}=\left[\begin{array}{ccc} 0.58 & 0.30 & 0.48\\ 0.30 & 0.71 & 0.37\\ 0.48 & 0.37 & 0.77 \end{array}\right]\text{.}$$

We consider various designs for our clinical trial based on choosing different follow-up periods (i.e., 2, 3, 4, 5, and 6 years) and assuming that it is of interest to detect minimally clinically meaningful treatment effects corresponding to 25% reductions in the annual rates of change in the MMSE, CDR-SB, and ADAS-11 (transformed). These 25% reductions here also correspond approximately to 25% improvements in the treated versus control arms, if the components were considered on their original scales of measurement.

### 2.4 Power analysis–specifying the weights

We compare various weights for Ξ_*JC*_(*w*) and Ξ_*C*_(*w*) (optimal or otherwise) that can be used when performing a power analysis for the clinical trial designs mentioned in the early subsection. All the considered weight vectors are normalized by $\sum_{j=1}^{J}w_{j}^{2}=1$. The following weighting strategies are considered:

1 The equal weights vector $w_{Z}={\left(3^{-1/2},3^{-1/2},3^{-1/2}\right)}^{\text{T}}$ assumes that the component treatment effects are equally important or that the treatment effect on the average of the component scores is of interest. Typically this strategy may be adopted in practice and therefore provides a benchmark to compare the other weighting strategies.
2 The unit vectors *w*_(1)_ = (1,0,0)^T^, *w*_(2)_ = (0,1,0)^T^, and *w*_(3)_ = (0,0,1)^T^ consider the situations in which either only one of the component treatment effects or the treatment effect on a single component is of interest.
3 The optimal weights vector for Ξ_*JC*_(*w*), denoted by $w_{JC}^{\text{*}}$, is optimal in the sense that ${\text{Ξ}}_{JC}\left(w_{JC}^{\text{*}}\right)$ has the greatest power to reject ${H^{\prime }}_{0}$ under a given alternative. In the Supplementary Materials, it is proven that ${\text{Ξ}}_{JC}\left(w_{JC}^{\text{*}}\right)$ is always more powerful than Ξ_*J*_ in rejecting the associated null hypothesis given same conditions. The optimal weights $w_{JC}^{\text{*}}$ are the eigenvector associated with the largest eigenvalue of $\sum_{\gamma }^{-1}\gamma^{\text{*}}\gamma^{\text{*T}}$, which is proportional to $\sum_{\gamma }^{-1}\gamma^{\text{*}}$, where $\gamma^{\text{*}}$ is the treatment effect vector under the alternative. In Table 2, we list the optimal weights for Ξ_*JC*_(*w*) for the different trial duration scenarios.

**Table 2:** The optimal weights for Ξ_*JC*_(*w*) and Ξ_*C*_(*w*) in each trial duration

Weights	Component	Trial duration				
		2 years	3 years	4 years	5 years	6 years
$w_{JC}^{\text{*}}$	MMSE	0.7670	0.7641	0.7576	0.7511	0.7451
	CDR-SB	0.4961	0.4958	0.4964	0.4971	0.4978
	ADAS-11	0.4069	0.4128	0.4238	0.4344	0.4438
$w_{C}^{\text{*}}$	MMSE	0.7151	0.7104	0.7061	0.7026	0.6999
	CDR-SB	0.5052	0.5050	0.5048	0.5046	0.5044
	ADAS-11	0.4832	0.4902	0.4966	0.5017	0.5057
4 The optimal weights vector for Ξ_*C*_(*w*), denoted by $w_{C}^{\text{*}}$, maximizes the power of Ξ_*C*_(*w*) to detect the treatment effects under a given alternative over all possible normalized *w*; see the Supplementary document for the algorithm to calculate $w_{C}^{\text{*}}$. The composite score induced by $w_{C}^{\text{*}}$ is the most sensitive for detecting a treatment effect on the composite score. The optimal weights $w_{C}^{\text{*}}$ for different trial scenarios are listed in Table 2.

## 3 Results

Table 3 presents the sample sizes required for each of the aforementioned weighting specifications and under the different trial duration scenarios when the statistical power is specified at 80% and the significance level is set at 5%. Also reported are the calculated sample sizes when each component is considered separately for powering the trial, and a Bonferroni correction is applied. Here, the maximum of the three calculated sample sizes based on the three components is chosen as the sample size to be specified for the trial.

**Table 3:** The sample sizes calculated by each approach with 80% statistical power and 5% significance level by trial duration

Test statistic	Weights	Trial duration				
		2 years	3 years	4 years	5 years	6 years
Ξ_*J*_	-	23,714	7041	2983	1550	908
Ξ_*JC*_(*w*)	*w*_(1)_	24,934	7447	3192	1678	994
	*w*_(2)_	45,259	13,548	5789	3030	1786
	*w*_(3)_	45,844	13,635	5789	3014	1769
	*w*_*Z*_	17,672	5242	2216	1149	672
	$w_{JC}^{\text{*}}$	**17,072**	**5069**	**2148**	**1116**	**654**
	$w_{C}^{\text{*}}$	17,139	5090	2156	1120	656
Ξ_*C*_(*w*)	*w*_(1)_	26,851	8059	3451	1809	1067
	*w*_(2)_	46,524	13,929	5943	3105	1827
	*w*_(3)_	47,654	14,189	6017	3126	1831
	$w_{Z}$	17,881	5306	2242	1162	679
	$w_{JC}^{\text{*}}$	17,625	5236	2214	1147	671
	$w_{C}^{\text{*}}$	17,549	5212	2205	1143	669
		2 years	3 years	4 years	5 years	6 years
Bonferroni correction		63,563	18,926	8025	4170	2443

NOTE. Numbers given in bold indicates the test statistic $\Xi_{JC}\left(w_{JC}^{*}\right)$ that gives the smallest sample sizes for each of the considered clinical trial design scenarios.

From the table, we observe that the test statistic $\Xi_{JC}\left(w_{JC}^{*}\right)$ gives the smallest sample sizes (numbers highlighted in bold) for each of the clinical trial design scenarios considered. Moreover, we make the following points after examining Table 3.

A substantial number of participants may be required when a trial for early AD only lasts for 2 years, under our assumptions. We estimate that at least 17,000 participants would need to be recruited in a 2-year AD trial in an MCI population to have sufficient power (i.e., 80%) to detect a 25% reduction in the annual rate of change on each of the transformed component scores. Recruitment of such numbers may be infeasible for a 2-year duration clinical trial in early AD with four biannual follow-up visits and even if feasible failure rates could potentially be high for early AD populations. Note that the required sample sizes will decrease with increasing trial duration, assuming biannual visits.

The required sample sizes to detect the treatment effect on the transformed MMSE are much smaller than the ones to detect the treatment effect on the transformed CDR-SB or ADAS-11 (comparing *w*_(1)_ rows to *w*_(2)_ and *w*_(3)_ rows in Table 3). Let us consider a clinical trial of 3 years duration as an example. The required sample sizes obtained by Ξ_*JC*_(*w*_(1)_) is 55.0% of the ones obtained by Ξ_*JC*_(*w*_(2)_) and 54.6% of the ones obtained by Ξ_*JC*_(*w*_(3)_). This implies that the transformed MMSE is the more sensitive measure for detecting a treatment effect for early AD than transformed CDR-SB and the ADAS-11 measures [15], [16], [17].

The approaches that use the optimal weights could require at least 60% fewer participants than the ones using *w*_(2)_ or *w*_(3)_. In our analysis, the performances of Ξ_*JC*_(*w*) and Ξ_*C*_(*w*) with *w*_*Z*_ are comparable to the ones using the optimal weights. This is a consequence of the estimated parameters obtained from the analysis of the ADNI data giving rise to optimal weights that are close to *w*_*Z*_ (Table 2). Comparable performances across these three statistics will not in general be expected when using other component outcomes.

The sample sizes calculated under Ξ_*JC*_(*w*) are always smaller than the ones calculated under Ξ_*C*_(*w*) for fixed weights, although the reduction may not be significant; for example, there is a 3% reduction in sample sizes when Ξ_*JC*_(*w*) is used with $w=w_{JC}^{*}$. Such gain in efficiency is obtained by specifying the correlation structure among the component scores in the MLMM.

## 4 Discussion

We have described three approaches for performing power analysis to detect treatment effects in clinical trials for early AD. From our investigations, we found that jointly modeling the component scores and then constructing sensitive test statistics or composite scores based on optimal weights will improve the efficiency of clinical trials. Under our model assumptions, testing based on the optimal composite treatment effect will lead to the smallest required sample sizes and therefore should be recommended when powering clinical trials in AD if treatment effects on multiple components are of interest.

We end the article with the following discussion points.

### 4.1 Model assumptions

We assume that the component scores are jointly from an MLMM. This may be too strong an assumption for analyzing some cognitive and function scores in AD, because the component scores usually are discrete with strong ceiling or floor effects. Consider the CDR-SB as an example. The CDR-SB is the sum of six component scores, including the Memory Score, the Orientation Score, the Judgement and Problem Solving Score, the Community Affairs Score, the Home and Hobbies Score, and the Personal Care Score. The component scores except the Personal Care Score have the discrete range 0, 0.5, 1, 2, and 3, whereas the Personal Care Score has the range 0, 1, 2, and 3. From the ADNI data, over 30% of individuals have 0 in each component score of the CDR-SB, which would indicate strong floor effects (zero-heavy data). Therefore, it may not be appropriate to use an MLMM with CDR-SB on its original scale or even after transformation as done in this article. The use of other models, which take account of zero-heavy data may be appropriate; see Farewell et al. [18] for a comprehensive review.

In our power analysis results, we took the covariance matrices of $\varepsilon_{nt}$ and *b*_*n*_ to be known when fitting the MLMM. This allowed us to obtain explicit formulas for the MLEs and their covariance, which enabled us to compare the powers of the test statistics and calculate the optimal composite scores. In practice, these covariance matrices would need to be estimated. They may be obtained from previous investigations or through a pilot study. However, note that without considering the variability in the estimated covariance matrices, there would be a tendency to underestimate the required sample sizes. Monte Carlo studies can be applied to obtain more accurate sample sizes [19]. However, these would require intensive computational work to compute the optimal weights.

In the MLMM for component scores, it is assumed that, for each *n*, the errors $\varepsilon_{nt}$, *t* = 1,…,*T*_*n*_, are independent across time. This implies that the time correlation of *Y*_*nt*_, *t* = 1,…,*T*_*n*_, is induced only through the random intercepts *b*_*n*_. This can be generalized so as to introduce the auto correlations between $\varepsilon_{nt}$, *t* = 1,…,*T*_*n*_. Such generalization would raise computational challenges, and a bespoke program would be needed. (We were unable to find a statistical software package that would allow us to fit this more generalized model).

### 4.2 Wald statistics

The considered Wald statistics are used to detect the component treatment effect, but they do not make distinction between beneficial effects and deleterious effects. However, because currently in early AD, there may be an expectation that any treatment brought forward for confirmatory testing in a phase III trial has undergone rigorous assessment at phase II to ensure that it does not confer harm, it may be of interest to investigate rejecting *H*_0_ under the alternative that all the component treatment effects γ are non-negative. In this situation, the Wald statistic Ξ_*J*_ follows a mixture of $\chi_{p}^{2}$ distribution, *P* = 0,…,*J*, where $\chi_{0}^{2}$ distribution is the distribution with mass 1 at point 0. In general, it is challenging to calculate the weights that combine the $\chi_{p}^{2}$ distribution, *P* = 0,…,*J*, [20].

When the weights *w* in Ξ_*JC*_(*w*) and Ξ_*C*_(*w*) are non-negative elementwise, we may modify the alternatives against ${H^{\prime }}_{0}$ and ${H^{\prime \prime }}_{0}$ to

$${H^{\prime }}_{A}:\sum_{j=1}^{J}w_{j}\gamma_{j}>0$$

and

$${H^{\prime \prime }}_{A}:\gamma_{w}>0\text{,}$$

respectively. We can use the *Z*-statistics, $\Xi_{JC}^{1/2}\left(w\right)$ and $\Xi_{C}^{1/2}\left(w\right)$, for the one-sided tests. They follow the standard normal distribution under their associated null hypothesis. However, the elements of the optimal weights $w_{JC}^{\text{*}}$ and $w_{C}^{\text{*}}$ may not always be non-negative.

### 4.3 Parameters necessary for powering clinical trials

It is crucial to obtain plausible values of the parameters needed for the power analysis, including the annual change rates, the covariance matrix of random effects, and the covariance matrix of errors. These parameter values can be informed from a pilot study or existing studies [21]. However, there always exists the concern whether the specified alternative truly represents the clinical trial target population effect of interest and how the variability of the alternatives will affect the calculated sample sizes, sensitivity analysis is recommended [4]. McEvoy et al. [22] compute 95% CIs on the sample sizes through bootstrapping. We also present the 95% bootstrap CIs for the calculated sample sizes in our Supplementary document.

The effect sizes must be determined based on rationale and justification from theory and clinical experiences [4]. When the effect sizes are set to be the percentages of the annual rate of change, they are approximately invariant to the transformation on the component scores if the term $\gamma_{j}\times \left(\text{Treatment}\times \text{Time}\right)+\beta_{j2}\times \text{Time}$ in the MLMM is around zero.

The derivation and use of optimal weights $w_{JC}^{\text{*}}$ and $w_{C}^{\text{*}}$ here were for the clinical purpose of powering a trial. We did not propose a new composite score to be used as an endpoint but constructed the most powerful test statistics with the optimal weights $w_{JC}^{\text{*}}$ and the most sensitive composite score with the weights $w_{C}^{\text{*}}$ to detect treatment effects. We further argued that no extra information or no further model assumption than what is typically needed is required to calculate them given the alternatives. Therefore, it is helpful to compute and use the optimal weights in power analysis. For other clinical purposes, the optimal weights *w* as defined and clinically meaningful weights may conflict. In such situations, we suggest modifying the criterion for determining the optimal weights to take account clinical meaningfulness.

**Research in Context**

1. Systematic review: The authors reviewed the literature on constructing composite scores sensitive to the early changes in cognition and function and for detecting treatment effects in clinical trials for early AD. Under the assumption that the component scores are jointly from an MLMM, three approaches are compared with regard to their power to detect treatment effects. The authors calculate sample sizes based on these three approaches.
2. Interpretation: Jointly modeling the component scores and using data-driven optimal weights will improve the efficiency of clinical trials for early AD. Power analysis based on using the optimal composite treatment effect requires the smallest sample sizes.
3. Future directions: It is required to study more flexible statistical models and develop associated software to power a study for early AD.
