# Supplementary material for: Power analysis to detect treatment effects in longitudinal clinical trials for Alzheimer's disease
Source: Alzheimers Dement (N Y). 2017 May 24;3(3):360–6. doi: 10.1016/j.trci.2017.04.007 (PMC5590710; doi:10.1016/j.trci.2017.04.007)
Supplement: Supplementary Material [file mmc1.pdf]

Supplementary Documents

Zhiyue Huang[1], Graciela Muniz-Terrera[2], and Brian D M Tom[1]

[1]MRC Biostatistics Unit, University of Cambridge, UK
[2]Centre for Dementia Prevention, University of Edinburgh, UK
May 10, 2017

1 Theoretical Results

Model Specifications

In this section, we formalize the MLMM for component scores as follows. For the t-th visit of the n-th independent participant, we observe a $J-$dimensional vector of the component scores $\boldsymbol{Y}_{nt}$, a $p \times J$ matrix of the fixed effect covariates $\boldsymbol{x}_{nt}$, and a $q-$dimensional vector of the random effect covariates $\boldsymbol{z}_{nt}$, $n = 1, \ldots, N$ and $t = 1, \ldots, T_n$. We also let the first element of $\boldsymbol{x}_{nt}$ be associated with the treatment effects. Let $\boldsymbol{X}_{nt} = \boldsymbol{I}_J \otimes \boldsymbol{x}_{nt}$ be a $Jp \times J$ matrix of the fixed effect covariates, $\boldsymbol{Z}_{nt} = \boldsymbol{I}_J \otimes \boldsymbol{z}_{nt}$ be a $Jq \times J$ matrix of the random effect covariates, where $\otimes$ is the Kronecker product. Further for each n, let $\boldsymbol{Y}_n = [\boldsymbol{Y}_{n1}^{\mathrm{T}}, \ldots, \boldsymbol{Y}_{nT_n}^{\mathrm{T}}]^{\mathrm{T}}$ be the JT_n-dimensional vector of the component scores, $\boldsymbol{X}_n = [\boldsymbol{X}_{n1}, \ldots, \boldsymbol{X}_{nt}]$ be the $Jp \times JT_n$ matrix of the fixed effect covariates and $\boldsymbol{Z}_n = [\boldsymbol{Z}_{n1}, \ldots, \boldsymbol{Z}_{nT_n}]$ be the $q \times JT_n$ matrix of the random effect covariates.

The regression parameter $\boldsymbol{\beta} = (\boldsymbol{\beta}_1^{\mathrm{T}}, \ldots, \boldsymbol{\beta}_J^{\mathrm{T}})^{\mathrm{T}}$ is a Jp-dimensional vector, where for each j, $\boldsymbol{\beta}_j$ is a $p-$dimensional vector and its second element, denoted by γ_j, is the treatment effect on the j-th component. The treatment effect vector $\boldsymbol{\gamma} = (\gamma_1, \ldots, \gamma_J)^{\mathrm{T}}$ is $J-$dimensional. Further let $\boldsymbol{b}_n = (\boldsymbol{b}_{n1}^{\mathrm{T}}, \ldots, \boldsymbol{b}_{nJ}^{\mathrm{T}})^{\mathrm{T}}$ be the $Jq-$dimensional vector of the random effects for the n-th participant, where for each j, $\boldsymbol{b}_{nj}$ is a $q-$dimensional vector of the random effects on the j-th component. It is further assumed that, for each n, the random effects $\boldsymbol{b}_n$ is independent to $\boldsymbol{X}_n$ and $\boldsymbol{Z}_n$.

For each n, the MLMM for component scores is

$$\boldsymbol{Y}_n = \boldsymbol{X}_n^{\mathrm{T}}\boldsymbol{\beta} + \boldsymbol{Z}_n^{\mathrm{T}}\boldsymbol{b}_n + \boldsymbol{\epsilon}_n,$$

where $\boldsymbol{b}_n \sim \text{MVN}(\mathbf{0}, \boldsymbol{\Sigma}_{\boldsymbol{b}})$ and $\boldsymbol{\epsilon}_n \sim \text{MVN}(0, \boldsymbol{I}_n \otimes \boldsymbol{\Sigma}_{\boldsymbol{\epsilon}})$. For each n, $\boldsymbol{I}_n$ is the $T_n \times T_n$ identity matrix. We assume that $\boldsymbol{\epsilon}_n$, $n = 1, \ldots, N$, are independent to each other. The covariance matrix of $\boldsymbol{\epsilon}_n = \boldsymbol{I}_n \otimes \boldsymbol{\Sigma}_e$ implies that the component scores $\boldsymbol{Y}_{nt}$, $t = 1, \ldots, T_n$, are independent to each other conditional on the random effects $\boldsymbol{b}_n$. Given a random sample from the considered MLMM and the true covariance matrices $\boldsymbol{\Sigma}_e$ and $\boldsymbol{\Sigma}_b$, we can obtain the maximum likelihood estimator (MLE) of $\boldsymbol{\beta}$, denoted by $\hat{\boldsymbol{\beta}}$, and its covariance matrix $\boldsymbol{\Sigma}_{\boldsymbol{\beta}}$. The MLE of the treatment effect vector, denoted by $\hat{\boldsymbol{\gamma}}$, is $\boldsymbol{E}^{\mathrm{T}}\hat{\boldsymbol{\beta}}$ with the covariance matrix $\boldsymbol{\Sigma}_{\boldsymbol{\gamma}} = \boldsymbol{E}^{\mathrm{T}}\boldsymbol{\Sigma}_{\boldsymbol{\beta}}\boldsymbol{E}$, where $\boldsymbol{E} = \boldsymbol{I}_J \otimes \boldsymbol{e}$ and $\boldsymbol{e} = (0, 1, 0, \ldots, 0)^{\mathrm{T}}$ is a $p-$dimensional vector.

Given any $J-$dimensional composite weights $\boldsymbol{w}$ with the constraint that $\|\boldsymbol{w}\|_2 = 1$, we can derive a LMM for the composite scores from the MLMM for component scores. For each n, the LMM is

$$\boldsymbol{C}_n = \boldsymbol{X}_{cn}^{\mathrm{T}}\boldsymbol{\beta}_w + \boldsymbol{Z}_{cn}^{\mathrm{T}}\boldsymbol{a}_n + \boldsymbol{\delta}_n,$$

where $\boldsymbol{C}_n = (\boldsymbol{I}_{T_n} \otimes \boldsymbol{w}^{\mathrm{T}})\boldsymbol{Y}_n$, $\boldsymbol{X}_{cn} = [\boldsymbol{x}_{n1}, \ldots, \boldsymbol{x}_{nT_n}]$, $\boldsymbol{\beta}_w = (\boldsymbol{w} \otimes \boldsymbol{I}_p)\boldsymbol{\beta}$, $\boldsymbol{Z}_{cn} = [\boldsymbol{z}_{n1}, \ldots, \boldsymbol{z}_{nT_n}]$, $\boldsymbol{a}_n = (\boldsymbol{w} \otimes \boldsymbol{I}_p)\boldsymbol{b}_n$ and $\boldsymbol{\delta}_n = (\boldsymbol{I}_{T_n} \otimes \boldsymbol{w}^{\mathrm{T}})\boldsymbol{\epsilon}_n$. The second element of $\boldsymbol{\beta}_w$ is $\gamma_w = \boldsymbol{w}^{\mathrm{T}}\boldsymbol{\gamma}$. We call it the composite treatment effect. Given the same random sample and the true covariance matrices $\boldsymbol{\Sigma}_e$ and $\boldsymbol{\Sigma}_b$, the MLE of $\boldsymbol{\beta}_w$ is denoted by $\hat{\boldsymbol{\beta}}_w$ with covariance matrix $\boldsymbol{\Sigma}_{\beta_w}$. Furthermore, the MLE of the composite treatment effect is $\hat{\gamma}_w = \boldsymbol{e}^{\mathrm{T}}\hat{\boldsymbol{\beta}}_w$ with variance $\sigma_\gamma^2 = \boldsymbol{e}^{\mathrm{T}}\boldsymbol{\Sigma}_{\beta_w}\boldsymbol{e}$.

Proof of Theorem 1

Theorem 1. *Given data from the MLMM for components, the test statistic $\Xi_C(\boldsymbol{w})$ has no more power than $\Xi_{JC}(\boldsymbol{w})$ for rejecting the null hypothesis H_0 for any composite $\boldsymbol{w} \in \mathrm{R}^J$ with $\|\boldsymbol{w}\|_2 = 1$. The two test statistics have the same power, when, for each n, there exists a $T_n \times T_n$ orthogonal matrix $\boldsymbol{P}_n$ such that each column of $\boldsymbol{W}_n\boldsymbol{P}_n$ is an eigenvector of $\boldsymbol{V}_n$, where for each n, $\boldsymbol{V}_n = \boldsymbol{I}_{T_n} \otimes \boldsymbol{\Sigma}_e + \boldsymbol{Z}_n^{\mathrm{T}}\boldsymbol{\Sigma}_b\boldsymbol{Z}_n$ and $\boldsymbol{W}_n = \boldsymbol{w} \otimes \boldsymbol{I}_p$.*

Proof. Because both of $\hat{\gamma}_w$ and $\boldsymbol{w}^{\mathrm{T}}\hat{\boldsymbol{\gamma}}$ are unbiased, we show that $\Xi_{JC}(\boldsymbol{w})$ is more efficient than $\Xi_C(\boldsymbol{w})$ by showing that

$$\boldsymbol{w}^{\mathrm{T}}\boldsymbol{E}^{\mathrm{T}}\boldsymbol{\Sigma}_{\boldsymbol{\beta}}\boldsymbol{E}\boldsymbol{w} \leq \boldsymbol{e}^{\mathrm{T}}\boldsymbol{\Sigma}_{\beta_w}\boldsymbol{e}$$

for any $\boldsymbol{w} \in \mathrm{R}^J$. Let $\boldsymbol{L} = \boldsymbol{w} \otimes \boldsymbol{I}_p$. Note that $\boldsymbol{L}^{\mathrm{T}}\boldsymbol{L} = \boldsymbol{I}$, and for each n, it is true that $\boldsymbol{X}_n\boldsymbol{W}_n = \boldsymbol{L}\boldsymbol{X}_{cn}$ and $\boldsymbol{L}\boldsymbol{e} = \boldsymbol{E}\boldsymbol{w}$.

According to the asymptotic results, we have

$$\boldsymbol{\Sigma}_{\boldsymbol{\beta}} = \left(\sum_{n=1}^{N} \boldsymbol{X}_n\boldsymbol{V}_n^{-1}\boldsymbol{X}_n^{\mathrm{T}}\right)^{-1}$$

and

$$\boldsymbol{\Sigma}_{\beta_w} = \left(\sum_{n=1}^{N} \boldsymbol{X}_{cn} \left(\boldsymbol{W}_n^{\mathrm{T}} \boldsymbol{V}_n \boldsymbol{W}_n \right)^{-1} \boldsymbol{X}_{cn}^{\mathrm{T}} \right)^{-1}.$$

For any $\boldsymbol{w} \in \mathrm{R}^J$, we have

$$\begin{aligned}
& \boldsymbol{w}^{\mathrm{T}} \boldsymbol{E}^{\mathrm{T}} \boldsymbol{\Sigma}_{\beta} \boldsymbol{E} \boldsymbol{w} \\
= \; & \boldsymbol{w}^{\mathrm{T}} \boldsymbol{E}^{\mathrm{T}} \left(\sum_{n=1}^{N} \boldsymbol{X}_n \boldsymbol{V}_n^{-1} \boldsymbol{X}_n^{\mathrm{T}} \right)^{-1} \boldsymbol{E} \boldsymbol{w} \\
= \; & \boldsymbol{e}^{\mathrm{T}} \boldsymbol{L}^{\mathrm{T}} \left(\sum_{n=1}^{N} \boldsymbol{X}_n \boldsymbol{V}_n^{-1} \boldsymbol{X}_n^{\mathrm{T}} \right)^{-1} \boldsymbol{L} \boldsymbol{e} \\
= \; & \boldsymbol{e}^{\mathrm{T}} \boldsymbol{L}^{\mathrm{T}} \left(\sum_{n=1}^{N} \boldsymbol{X}_n \boldsymbol{V}_n^{-1/2} \boldsymbol{V}_n^{-1/2} \boldsymbol{X}_n^{\mathrm{T}} \right)^{-1} \boldsymbol{L} \boldsymbol{e} \\
\leq \; & \boldsymbol{e}^{\mathrm{T}} \boldsymbol{L}^{\mathrm{T}} \left(\sum_{n=1}^{N} \boldsymbol{X}_n \boldsymbol{V}_n^{-1/2} \boldsymbol{V}_n^{1/2} \boldsymbol{W}_n \left(\boldsymbol{W}_n^{\mathrm{T}} \boldsymbol{V}_n \boldsymbol{W}_n \right)^{-1} \boldsymbol{W}_n^{\mathrm{T}} \boldsymbol{V}_n^{1/2} \boldsymbol{V}_n^{-1/2} \boldsymbol{X}_n^{\mathrm{T}} \right)^{-1} \boldsymbol{L} \boldsymbol{e} \\
= \; & \boldsymbol{e}^{\mathrm{T}} \boldsymbol{L}^{\mathrm{T}} \left(\sum_{n=1}^{N} \boldsymbol{X}_n \boldsymbol{W}_n \left(\boldsymbol{W}_n^{\mathrm{T}} \boldsymbol{V}_n \boldsymbol{W}_n \right)^{-1} \boldsymbol{W}_n^{\mathrm{T}} \boldsymbol{X}_n^{\mathrm{T}} \right)^{-1} \boldsymbol{L} \boldsymbol{e} \\
= \; & \boldsymbol{e}^{\mathrm{T}} \boldsymbol{L}^{\mathrm{T}} \left(\sum_{n=1}^{N} \boldsymbol{L} \boldsymbol{X}_{cn} \left(\boldsymbol{W}_n^{\mathrm{T}} \boldsymbol{V}_n \boldsymbol{W}_n \right)^{-1} (\boldsymbol{L} \boldsymbol{X}_{cn})^{\mathrm{T}} \right)^{-1} \boldsymbol{L} \boldsymbol{e}.
\end{aligned}$$

The inequality follows from the Cauchy-Schwarz inequality for matrices

$$\boldsymbol{A}\boldsymbol{A}^{\mathrm{T}} \geq \boldsymbol{A}\boldsymbol{B}^{\mathrm{T}} (\boldsymbol{B}\boldsymbol{B}^{\mathrm{T}})^{-1} \boldsymbol{B}\boldsymbol{A}^{\mathrm{T}}.$$

The equality holds if and only if for each n, there exists a $T_n \times T_n$ orthogonal matrix $\boldsymbol{P}_n$ such that each column of $\boldsymbol{W}_n \boldsymbol{P}_n$ is an eigenvector of $\boldsymbol{V}_n$.

Let $\boldsymbol{U}$ be the matrix of the eigenvectors of $\sum_{n=1}^{N} \boldsymbol{L}\boldsymbol{X}_{cn} (\boldsymbol{W}_n^{\mathrm{T}} \boldsymbol{V}_n \boldsymbol{W}_n)^{-1} (\boldsymbol{L}\boldsymbol{X}_{cn})^{\mathrm{T}}$, i.e.,

$$\sum_{n=1}^{N} \boldsymbol{L} \boldsymbol{X}_{cn} \left(\boldsymbol{W}_n^{\mathrm{T}} \boldsymbol{V}_n \boldsymbol{W}_n \right)^{-1} \boldsymbol{X}_{cn}^{\mathrm{T}} \boldsymbol{L}^{\mathrm{T}} \boldsymbol{U}^{\mathrm{T}} = \boldsymbol{U}^{\mathrm{T}} \boldsymbol{\Lambda},$$

where $\boldsymbol{\Lambda}$ is the diagonal matrix of the eigenvalues. We also have that

$$\left(\sum_{n=1}^{N} \boldsymbol{X}_{cn} \left(\boldsymbol{W}_n^{\mathrm{T}} \boldsymbol{V}_n \boldsymbol{W}_n \right)^{-1} \boldsymbol{X}_{cn}^{\mathrm{T}} \right)^{-1} \boldsymbol{L}^{\mathrm{T}} \boldsymbol{U}^{\mathrm{T}} = \boldsymbol{L}^{\mathrm{T}} \boldsymbol{U}^{\mathrm{T}} \boldsymbol{\Lambda}^{-1},$$

It follows that

$$\begin{aligned}
\boldsymbol{w}^{\mathrm{T}}\boldsymbol{E}^{\mathrm{T}}\boldsymbol{\Sigma}_{\beta}\boldsymbol{E}\boldsymbol{w} &\leq \boldsymbol{e}^{\mathrm{T}}\boldsymbol{L}^{\mathrm{T}}\left(\sum_{n=1}^{N}\boldsymbol{L}\boldsymbol{X}_{cn}\left(\boldsymbol{W}_n^{\mathrm{T}}\boldsymbol{V}_n\boldsymbol{W}_n\right)^{-1}(\boldsymbol{L}\boldsymbol{X}_{cn})^{\mathrm{T}}\right)^{-1}\boldsymbol{L}\boldsymbol{e} \\
&= \boldsymbol{e}^{\mathrm{T}}\boldsymbol{L}^{\mathrm{T}}\boldsymbol{U}^{\mathrm{T}}\boldsymbol{\Lambda}^{-1}\boldsymbol{U}\boldsymbol{L}\boldsymbol{e} \\
&= \boldsymbol{e}^{\mathrm{T}}\left(\sum_{n=1}^{N}\boldsymbol{X}_{cn}\left(\boldsymbol{W}_n^{\mathrm{T}}\boldsymbol{V}_n\boldsymbol{W}_n\right)^{-1}\boldsymbol{X}_{cn}^{\mathrm{T}}\right)^{-1}\boldsymbol{e} \\
&= \boldsymbol{e}^{\mathrm{T}}\boldsymbol{\Sigma}_{\beta_w}\boldsymbol{e}.
\end{aligned}$$

□

Proof of Theorem 2

Theorem 2. *Given the significance level α and power value, the test statistic Ξ_{JC}^* is always more powerful than Ξ_J.*

Proof. Let N_{JC} be the sample size calculated by $\Xi_{JC}(\boldsymbol{w})$. Given the significance level α and *power*,

$$N_{JC} = d_{JC}\sigma^2_{\boldsymbol{w}^{\mathrm{T}}\boldsymbol{\gamma}}(\boldsymbol{w}^{\mathrm{T}}\boldsymbol{\gamma}^*)^{-2},$$

where

$$\Pr(\Xi_{JC} \geq \psi_1(\alpha)) = power,$$

and Ξ_{JC} follows the noncentral χ^2 distribution with degrees of freedom 1 and the noncentrality parameter d_{JC}. When $\boldsymbol{w}^*(\Xi_{JC})$ is used,

$$N_{JC}^* = d_{JC}((\boldsymbol{\gamma}^*)^{\mathrm{T}}\boldsymbol{\Sigma}_{\boldsymbol{\gamma}}^{-1}\boldsymbol{\gamma}^*)^{-1},$$

because

$$\max(\boldsymbol{w}^{\mathrm{T}}\boldsymbol{\gamma}^*)^2\left(\boldsymbol{w}^{\mathrm{T}}\boldsymbol{\Sigma}_{\boldsymbol{\gamma}}\boldsymbol{w}\right)^{-1} = (\boldsymbol{\gamma}^*)^{\mathrm{T}}\boldsymbol{\Sigma}_{\boldsymbol{\gamma}}^{-1}\boldsymbol{\gamma}^*.$$

It follows that

$$N_{JC}^*(\boldsymbol{\gamma}^*)^{\mathrm{T}}\boldsymbol{\Sigma}_{\boldsymbol{\gamma}}^{-1}\boldsymbol{\gamma}^* = d_{JC}$$

On the other hand, let N_J be the sample size calculated by Ξ_J. Given the significance level α and *power*,

$$N_J = d_J((\boldsymbol{\gamma}^*)^{\mathrm{T}}\boldsymbol{\Sigma}_{\boldsymbol{\gamma}}^{-1}\boldsymbol{\gamma}^*)^{-1},$$

where

$$\Pr(\Xi_J \geq \psi_J(\alpha)) = power,$$

and Ξ_J follows the noncentral χ^2 distribution with degrees of freedom J and the noncentrality parameter d_J. Because $d_J \geq d_{JC}$, we have $N_J \geq N_{JC}^*$. In other words, Ξ_{JC}^* is more powerful than Ξ_J. □

2 MLMM for the ADNI Data

To make the component scores (the MMSE, the CDR-SB and the ADAS-11) in the ADNI dataset suitable to the model assumption, we use the following transformation

$$\begin{aligned} Y'_{nt1} &= -((Y''_{nt1}+1)^{-0.6}-1)/0.6, \\ Y'_{nt2} &= -((36-Y''_{nt2})^{-0.7}-1)/0.7, \end{aligned}$$

and

$$Y'_{nt3} = \log(Y''_{nt3}+1),$$

where Y''_{nt1}, Y''_{nt2} and Y''_{nt3} are the MMSE, the CDR-SB and the ADAS-11 of the nth individual at time t correspondingly. The Box-Cox transformation are applied on the CDR-SB and the MMSE with the transformation parameter $\lambda = -0.7$ and -0.6 correspondingly. The transformation parameters are estimated by maximizing profile log-likelihoods for the transformation parameter with the linear model

$$\begin{aligned} Y_{n0j} &= \beta_{j0} + \beta_{j1} \times \text{Baseline Dementia Status} \\ &\quad + \beta_{j2} \times \text{Age at Baseline} + \beta_{j3} \times \text{Marital Status} \\ &\quad + \beta_{j4} \times \text{Years of Education} + \epsilon_{nj}, \end{aligned}$$

where ϵ_{nj} follows a normal distribution and $j = 1, 2$. Next, the component scores are rescaled by dividing the standard deviation of their baseline values Y'_{n0j}, denoted by $sd(Y'_{n0j})$, i.e., for each $j \in \{1, 2, 3\}$,

$$Y_{ntj} = Y'_{ntj}/sd(Y'_{n0j}).$$

For each n, t and j, we consider the following model, say the MLMM (1),

$$\begin{aligned} Y_{ntj} &= \beta_{j0} + \beta_{j1} \times \text{Baseline Dementia Status} \\ &\quad + \beta_{j2} \times \text{Age at Baseline} + \beta_{j3} \times \text{Marital Status} \\ &\quad + \beta_{j4} \times \text{Years of Education} + \beta_{j5} \times \text{Years from Baseline} \\ &\quad + b_{nj} + \epsilon_{ntj}, \end{aligned}$$

where b_{nj} and ϵ_{ntj} are normal random variables. Let $\boldsymbol{b}_n = (b_{n1}, b_{n2}, b_{n3})^{\mathrm{T}} \in \mathrm{R}^3$, which follows a multivariate normal distribution $\mathrm{MVN}(\mathbf{0}, \boldsymbol{\Sigma}_b)$. Let $\boldsymbol{\epsilon}_n = (\boldsymbol{\epsilon}_{n1}^{\mathrm{T}}, \ldots, \boldsymbol{\epsilon}_{nT_n}^{\mathrm{T}})^{\mathrm{T}} \in \mathrm{R}^{3T_n}$, where for each t, $\boldsymbol{\epsilon}_{nt} = (\epsilon_{nt1}, \epsilon_{nt2}, \epsilon_{nt3})^{\mathrm{T}} \in \mathrm{R}^3$. For each n, $\boldsymbol{\epsilon}_n$ follows a multivariate normal distribution $\mathrm{MVN}(\mathbf{0}, \boldsymbol{I}_{T_n} \otimes \boldsymbol{\Sigma}_\epsilon)$, where $\otimes$ is the Kronecker product. The estimated regression parameters are summarized in Table 1. The estimated covariance matrices are

$$\hat{\boldsymbol{\Sigma}}_\epsilon = \begin{bmatrix} 0.56 & 0.07 & 0.09 \\ 0.07 & 0.57 & 0.06 \\ 0.09 & 0.06 & 0.44 \end{bmatrix} \quad \text{and} \quad \hat{\boldsymbol{\Sigma}}_b = \begin{bmatrix} 0.58 & 0.30 & 0.48 \\ 0.30 & 0.71 & 0.37 \\ 0.48 & 0.37 & 0.77 \end{bmatrix}.$$

The associated correlation matrices are

$$\hat{\boldsymbol{\rho}}_\epsilon = \begin{bmatrix} 1.00 & 0.13 & 0.19 \\ 0.13 & 1.00 & 0.12 \\ 0.19 & 0.12 & 1.00 \end{bmatrix} \quad \text{and} \quad \hat{\boldsymbol{\rho}}_b = \begin{bmatrix} 1.00 & 0.46 & 0.71 \\ 0.46 & 1.00 & 0.49 \\ 0.71 & 0.49 & 1.00 \end{bmatrix}.$$

We also consider the simple MLMM that, for each n, t and j,

$$Y_{ntj} = \beta_{j0} + \beta_{j5} \times \text{ Years from Baseline} + b_{nj} + \epsilon_{ntj}$$

where $\boldsymbol{b}_n = (b_{n1}, b_{n2}, b_{n3})^{\mathrm{T}} \in \mathrm{R}^3$ follows a multivariate normal distribution $\mathrm{MVN}(\mathbf{0}, \boldsymbol{\Sigma}_b)$ and $\boldsymbol{\epsilon}_n = (\boldsymbol{\epsilon}_{n1}^{\mathrm{T}}, \ldots, \boldsymbol{\epsilon}_{nT_n}^{\mathrm{T}})^{\mathrm{T}} \in \mathrm{R}^{3T_n}$ follows a multivariate normal distribution $\mathrm{MVN}(\mathbf{0}, \boldsymbol{I}_{T_n} \otimes \boldsymbol{\Sigma}_\epsilon)$, for each t, $\boldsymbol{\epsilon}_{nt} = (\epsilon_{nt1}, \epsilon_{nt2}, \epsilon_{nt3})^{\mathrm{T}} \in \mathrm{R}^3$. The estimated annual change rates are summarized in Table 2. The estimated covariance matrices are

$$\hat{\boldsymbol{\Sigma}}_\epsilon = \begin{bmatrix} 0.57 & 0.07 & 0.09 \\ 0.07 & 0.57 & 0.06 \\ 0.09 & 0.06 & 0.44 \end{bmatrix} \quad \text{and} \quad \hat{\boldsymbol{\Sigma}}_b = \begin{bmatrix} 0.75 & 0.42 & 0.68 \\ 0.42 & 0.81 & 0.52 \\ 0.68 & 0.52 & 1.02 \end{bmatrix},$$

and the associated correlation matrices are

$$\hat{\boldsymbol{\rho}}_\epsilon = \begin{bmatrix} 1.00 & 0.13 & 0.19 \\ 0.13 & 1.00 & 0.12 \\ 0.19 & 0.12 & 1.00 \end{bmatrix} \quad \text{and} \quad \hat{\boldsymbol{\rho}}_b = \begin{bmatrix} 1.00 & 0.53 & 0.76 \\ 0.53 & 1.00 & 0.57 \\ 0.76 & 0.57 & 1.00 \end{bmatrix}.$$

The estimates of the parameters for power analysis, β_{j5}, $j = 1, 2, 3$, $\boldsymbol{\Sigma}_\epsilon$ and $\boldsymbol{\Sigma}_b$, are close to the ones from the MLMM (1). In this paper, we use the estimates from the MLMM (1) as the parameter values for power analysis.

3 Sensitivity Analysis

To further investigate the variability of the considered alternatives, which are induced by fitting the ADNI data, we obtain a 95% bootstrap confidence interval of the calculated sample sizes; see Table 3. At each bootstrap replication, we sample the MCI participants from the ADNI data with replacement, obtain the alternatives by fitting the MLMM to the new sample, and use them to calculate the required sample sizes. Due to the intensive computational workload, the number of bootstrap replications is 100. With larger number of bootstrap replications , we may obtain more accurate confidence intervals. From Table 2, we can observe similar performances of the test statistics $\Xi_{JC}(\boldsymbol{w})$ and $\Xi_C(\boldsymbol{w})$ when $\boldsymbol{w} = \boldsymbol{w}_Z$, $\boldsymbol{w} = \boldsymbol{w}_{JC}^*$ and $\boldsymbol{w} = \boldsymbol{w}_C^*$. This is specific to the parameters in the considered alternatives.

	Estimate	STD	95% CI	p-value
MMSE				
Intercept	20.943	0.366	(20.225, 21.660)	<0.01
BaseLine Dementia Status	0.439	0.040	(0.360, 0.517)	<0.01
Age at Baseline	0.023	0.004	(0.015, 0.031)	<0.01
Martial Status	-0.082	0.036	(-0.151, -0.012)	0.022
Educated Years	-0.065	0.010	(-0.085, -0.045)	<0.01
Years from Baseline	0.079	0.008	(0.063, 0.095)	<0.01
CDR-SB				
Intercept	1.388	0.395	(0.613, 2.163)	<0.01
BaseLine Dementia Status	0.381	0.043	(0.297, 0.465)	<0.01
Age at Baseline	0.012	0.004	(0.004, 0.020)	<0.01
Martial Status	-0.155	0.038	(-0.230, -0.079)	<0.01
Educated Years	-0.019	0.011	(-0.040, 0.003)	0.086
Years from Baseline	0.061	0.008	(0.045, 0.077)	<0.01
ADAS-11				
Intercept	2.106	0.400	(1.321, 2.890)	<0.01
BaseLine Dementia Status	0.474	0.043	(0.390, 0.558)	<0.01
Age at Baseline	0.038	0.004	(0.029, 0.046)	<0.01
Martial Status	-0.130	0.039	(-0.206, -0.053)	<0.01
Educated Years	-0.052	0.011	(-0.074, -0.030)	<0.01
Years from Baseline	0.055	0.007	(0.041, 0.069)	<0.01

Table 1: Summary of Estimated Regression Parameters in the MLMM (1).

	Estimate	STD	95% CI	p-value
MMSE				
Intercept	23.034	0.034	(22.968, 23.100)	<0.01
Years from Baseline	0.071	0.007	(0.056, 0.087)	<0.01
CDR-SB				
Intercept	3.089	0.035	(3.021, 3.156)	<0.01
Years from Baseline	0.054	0.007	(0.039, 0.070)	<0.01
ADAS-11				
Intercept	5.5139	0.037	(5.442, 5.586)	<0.01
Years from Baseline	0.049	0.007	(0.036, 0.064)	<0.01

Table 2: Summary of Estimated Regression Parameters in the Simple MLMM.

Table 3: The 95% bootstrap confidence internval of the sample sizes calculated by each approach with 80% statistical power and 5% significance level in each trial duration.

Test Statistic	Weights	Trial Duration<br>2 years	3 years	4 years	5 years	6 years
Ξ_J	-	(22697 , 32956)	(6691 , 9712)	(2892 , 4194)	(1533 , 2222)	(888 , 1286)
$\Xi_{JC}(\boldsymbol{w})$	$\boldsymbol{w}_{(1)}$	(21048 , 30679)	(6173 , 9000)	(2670 , 3891)	(1420 , 2069)	(826 , 1203)
	$\boldsymbol{w}_{(2)}$	(29870 , 61450)	(8778 , 18069)	(3782 , 7792)	(2001 , 4125)	(1157 , 2387)
	$\boldsymbol{w}_{(3)}$	(34899 , 58325)	(10195 , 17026)	(4367 , 7289)	(2299 , 3836)	(1325 , 2209)
	$\boldsymbol{w}_Z$	(14604 , 21647)	(4253 , 6307)	(1813 , 2690)	(950 , 1411)	(545 , 810)
	$\boldsymbol{w}^*_{JC}$	(14013 , 20745)	(4083 , 6049)	(1743 , 2586)	(915 , 1359)	(526 , 781)
	$\boldsymbol{w}^*_C$	(14065 , 20824)	(4098 , 6074)	(1749 , 2595)	(918 , 1363)	(527 , 784)
$\Xi_C(\boldsymbol{w})$	$\boldsymbol{w}_{(1)}$	(22697 , 32956)	(6691 , 9712)	(2892 , 4194)	(1533 , 2222)	(888 , 1286)
	$\boldsymbol{w}_{(2)}$	(30710 , 63142)	(9026 , 18571)	(3883 , 7997)	(2051 , 4227)	(1184 , 2441)
	$\boldsymbol{w}_{(3)}$	(36270 , 60586)	(10605 , 17706)	(4536 , 7570)	(2384 , 3976)	(1370 , 2285)
	$\boldsymbol{w}_Z$	(14785 , 21885)	(4307 , 6377)	(1836 , 2719)	(962 , 1425)	(551 , 817)
	$\boldsymbol{w}^*_{JC}$	(14427 , 21396)	(4203 , 6243)	(1791 , 2663)	(938 , 1395)	(538 , 800)
	$\boldsymbol{w}^*_C$	(14370 , 21308)	(4187 , 6215)	(1784 , 2652)	(935 , 1391)	(536 , 798)

4 Computing $\boldsymbol{w}^*(\Xi_C)$

We have that

$$\begin{aligned} & \frac{\partial}{\partial \boldsymbol{w}}(\boldsymbol{w}^{\mathrm{T}}\boldsymbol{\gamma}^*)^2(\boldsymbol{e}^{\mathrm{T}}\boldsymbol{\Sigma}_{\boldsymbol{\beta}_w}\boldsymbol{e})^{-1} \\ = \;& \boldsymbol{\gamma}^*(\boldsymbol{\gamma}^*)^{\mathrm{T}}\boldsymbol{w}(\boldsymbol{e}^{\mathrm{T}}\boldsymbol{\Sigma}_{\boldsymbol{\beta}_w}\boldsymbol{e})^{-1} - (\boldsymbol{w}^{\mathrm{T}}\boldsymbol{\gamma}^*)^2(\boldsymbol{e}^{\mathrm{T}}\boldsymbol{\Sigma}_{\boldsymbol{\beta}_w}\boldsymbol{e})^{-2}\frac{\partial}{\partial \boldsymbol{w}}(\boldsymbol{e}^{\mathrm{T}}\boldsymbol{\Sigma}_{\boldsymbol{\beta}_w}\boldsymbol{e}). \end{aligned}$$

By setting the first order derivative to zero, we have the non-linear equation

$$0 = \boldsymbol{\gamma}^*(\boldsymbol{e}^{\mathrm{T}}\boldsymbol{\Sigma}_{\boldsymbol{\beta}_w}\boldsymbol{e}) - (\boldsymbol{w}^{\mathrm{T}}\boldsymbol{\gamma}^*)\frac{\partial}{\partial \boldsymbol{w}}(\boldsymbol{e}^{\mathrm{T}}\boldsymbol{\Sigma}_{\boldsymbol{\beta}_w}\boldsymbol{e}).$$

By Taylor's expansion locally at the solution $\boldsymbol{w}_0$, we have

$$\begin{aligned} 0 = \;& \left(\boldsymbol{\gamma}^*(\boldsymbol{e}^{\mathrm{T}}\boldsymbol{\Sigma}_{\boldsymbol{\beta}_w}\boldsymbol{e}) - (\boldsymbol{w}^{\mathrm{T}}\boldsymbol{\gamma}^*)\frac{\partial}{\partial \boldsymbol{w}}(\boldsymbol{e}^{\mathrm{T}}\boldsymbol{\Sigma}_{\boldsymbol{\beta}_w}\boldsymbol{e})\right)\Bigg|_{\boldsymbol{w}=\boldsymbol{w}_0} \\ & - \left((\boldsymbol{w}^{\mathrm{T}}\boldsymbol{\gamma}^*)\frac{\partial^2}{\partial \boldsymbol{w}\partial \boldsymbol{w}^{\mathrm{T}}}(\boldsymbol{e}^{\mathrm{T}}\boldsymbol{\Sigma}_{\boldsymbol{\beta}_w}\boldsymbol{e})\right)\Bigg|_{\boldsymbol{w}=\boldsymbol{w}_0}(\boldsymbol{w}-\boldsymbol{w}_0). \end{aligned}$$

Because it is hard to calculate matrix

$$\frac{\partial^2}{\partial \boldsymbol{w}\partial \boldsymbol{w}^{\mathrm{T}}}(\boldsymbol{e}^{\mathrm{T}}\boldsymbol{\Sigma}_{\boldsymbol{\beta}_w}\boldsymbol{e}),$$

we use the approximation

$$\boldsymbol{B}(\boldsymbol{w}) = \boldsymbol{e}^{\mathrm{T}}\boldsymbol{H}^{-1}\boldsymbol{X}_c\boldsymbol{G}^{-1}\left(\frac{\partial^2 \boldsymbol{G}}{\partial \boldsymbol{w}\partial \boldsymbol{w}^{\mathrm{T}}}\right)\boldsymbol{G}^{-1}\boldsymbol{X}_c^{\mathrm{T}}\boldsymbol{H}^{-1}\boldsymbol{e} \tag{1}$$

where

$$\begin{aligned} \boldsymbol{X}_c &= [\boldsymbol{X}_{c1}, \ldots, \boldsymbol{X}_{cN}], \\ \boldsymbol{H} &= \boldsymbol{X}_c\boldsymbol{G}^{-1}\boldsymbol{X}_c^{\mathrm{T}}, \\ \boldsymbol{G} &= \bigoplus_{n=1}^{N}\boldsymbol{W}_n^{\mathrm{T}}\left(\boldsymbol{I}_{T_n}\otimes\boldsymbol{\Sigma}_e + \boldsymbol{Z}_n^{\mathrm{T}}\boldsymbol{\Sigma}_b\boldsymbol{Z}_n\right)\boldsymbol{W}_n, \end{aligned}$$

and $\oplus$ is the direct sum of matrices. We describe the algorithm as follows. Note that this algorithm only give a local optimum solution (either maximum or minimum). And thus, the validation of the solution is required. The MATLAB code is provided in later sections.

Algorithm 1. *Given an initial $\boldsymbol{w}^{(0)}$, at the sth iteration, we repeat the following steps.*

1. *Update* $\boldsymbol{w}^{(s+1)}$ *by*

$$\boldsymbol{w}^{(s+1)} = \boldsymbol{w}^{(s)} + ((\boldsymbol{w}^{(s)})^{\mathrm{T}}\gamma^*)^{-1}\boldsymbol{B}^{-1}(\boldsymbol{w}^{(s)}) \left(\gamma^*(\boldsymbol{e}^{\mathrm{T}}\boldsymbol{\Sigma}_{\beta_w}\boldsymbol{e}) - ((\boldsymbol{w})^{\mathrm{T}}\gamma^*)\frac{\partial}{\partial \boldsymbol{w}}(\boldsymbol{e}^{\mathrm{T}}\boldsymbol{\Sigma}_{\beta_w}\boldsymbol{e})\right)\Bigg|_{\boldsymbol{w}=\boldsymbol{w}^{(s)}}.$$

2. *Normalize the vector* $\tilde{\boldsymbol{w}}^{(s+1)}$ *in* L_2*-norm, i.e.,* $\boldsymbol{w}^{(s+1)} = \tilde{\boldsymbol{w}}^{(s+1)}(\|\tilde{\boldsymbol{w}}^{(s+1)}\|_2)^{-1}$.
3. *Update* $s = s + 1$.
4. *Stop the iteration until* $\|\boldsymbol{w}^{(s)} - \boldsymbol{w}^{(s+1)}\|_2 < \epsilon$, *where* $\epsilon > 0$.

5 MATLAB Code for Computing $w^*(\Xi_C)$

```
function [wnew, obj] = opt_C(betat, Sigmab, SigmaY, GammaT,
    T, J, w0)

% Calculate the optimum weights for LMM.
% The covariance matrix of \hat{beta}_w
% is computed though by Monte-Carlo methods.

% The inputs of this function are
% betat: Regression parameter;
% Sigmab: Covariance matrix of random effects;
% SigmaY: Covariance matrix of errors;
% GammaT: Type of covariance structure among time;
% T:      Number of visits;
% J:      Number of sub-tests;
% w0:     Initial value of weights.

N0 = 2e2;
% Number of fixed covariates
p = length(betat)/J;
% Covariance matrix of errors
SIGMAe = kron(eye(T*N0), SigmaY);
% Covariance matrix of random effects
SIGMAd = kron(eye(N0),Sigmab);

e = zeros(p,1);
e(2) = 1;
E = kron(eye(J),e);
gammat = E'*betat;

Ind= 1;
while Ind == 1

    % Compute the matrices with w_0
    W = kron(eye(N0*T), w0);
    G = W'*(SIGMAe + Zd'*SIGMAd*Zd) *W;
```

```
    H = Xc*inv(G)*Xc';
    LP = e'*inv(H)*Xc*inv(G);

    dL = zeros(J,1); ddL = zeros(J);
    for j = 1:J
        ewj = zeros(1,J);
        ewj(j) = 1;
        % Compute the gradient
        dGj = kron(eye(N0*T),ewj)*...
              (SIGMAe+Zd'*SIGMAd*Zd)*W +...
              W'*(SIGMAe+Zd'*SIGMAd*Zd)*...
              kron(eye(N0*T), ewj');
        dL(j) = LP*dGj*LP'*(w0'*gammat);

        % Compute the approximated Hessian
        for i = 1:J
            ewi = zeros(1,J);
            ewi(i) = 1;
            dGji = kron(eye(N0*T), ewj)*...
                   (SIGMAe+Zd'*SIGMAd*Zd)*...
                   kron(eye(N0*T), ewi')+...
                   kron(eye(N0*T), ewi)*...
                   (SIGMAe+Zd'*SIGMAd*Zd)*...
                   kron(eye(N0*T), ewj');
            ddL(i,j)= LP*dGji*LP'*(w0'*gammat);
        end
    end
    dL = dL - gammat*(e'*inv(H)*e);

    % Solve the estimating equation
    wnew = w0 - inv(ddL)*dL;

    % Normalize w^{(s+1)}
    wnew = wnew/norm(wnew);

    % Stopping Criteria
    if  norm(wnew-w0) <1e-4
        Ind=0;
    else
        w0 = wnew;
    end

    % Compute the objective function value
    obj = (wnew'*gammat)^2*(e'*inv(H)*e)^(-1);
end
```
